# The first activation study of the β-carbonic anhydrases from the pathogenic bacteria *Brucella suis* and *Francisella tularensis* with amines and amino acids

**DOI:** 10.1080/14756366.2019.1630617

**Published:** 2019-07-08

**Authors:** Andrea Angeli, Sonia Del Prete, Mariana Pinteala, Stelian S. Maier, William A. Donald, Bogdan C. Simionescu, Clemente Capasso, Claudiu T. Supuran

**Affiliations:** a Neurofarba Department, Sezione di Scienze Farmaceutiche e Nutraceutiche, Università degli Studi di Firenze, Sesto Fiorentino, Italy;; b Istituto di Bioscienze e Biorisorse, CNR, Napoli, Italy;; c Centre of Advanced Research in Bionanoconjugates and Biopolymers Department, “Petru Poni” Institute of Macromolecular Chemistry, Iasi, Romania;; d Polymers Research Center, Polymeric Release Systems Research Group, “Gheorghe Asachi” Technical University of Iasi, Iasi, Romania;; e School of Chemistry, University of New South Wales, Sydney, Australia

**Keywords:** Carbonic anhydrase, metalloenzymes, bacteria, activators, *Brucella suis*

## Abstract

The activation of the β-class carbonic anhydrases (CAs, EC 4.2.1.1) from the bacteria *Brucella suis* and *Francisella tularensis* with amine and amino acids was investigated. BsuCA 1 was sensitive to activation with amino acids and amines, whereas FtuCA was not. The most effective BsuCA 1 activators were L-adrenaline and D-Tyr (K_A_s of 0.70–0.95 µM). L-His, L-/D-Phe, L-/D-DOPA, L-Trp, L-Tyr, 4-amino-L-Phe, dopamine, 2-pyridyl-methylamine, D-Glu and L-Gln showed activation constants in the range of 0.70–3.21 µM. FtuCA was sensitive to activation with L-Glu (K_A_ of 9.13 µM). Most of the investigated compounds showed a weak activating effect against FtuCA (K_A_s of 30.5–78.3 µM). Many of the investigated amino acid and amines are present in high concentrations in many tissues in vertebrates, and their role in the pathogenicity of the two bacteria is poorly understood. Our study may bring insights in processes connected with invasion and pathogenic effects of intracellular bacteria.

## 1. Introduction

The pathogenic bacteria *Brucella suis* ^1^ ^,^ ^2^ and *Francisella tularensis* ^3^ ^,^ ^4^ provoke serious diseases both in human and livestock, are difficult to treat by antibiotics, and have the potential to induce widespread infections. In fact, both *Brucella* and *Francisella* might be used as bioterrorism organisms due to the fact that quite low doses of pathogens (as few as 10–100 bacteria) are highly infectious, leading to ravaging epidemics^1^. Furthermore, they persist in the environment and are rapidly transmitted via different routes, including by aerosols and from human to human^1^. Brucellosis, like tularemia (the infection produced by *F. tularensis*) are neglected diseases, although their prevalence in humans and domestic/wild animals is not at all low^1–4^. These pathogens also became resistant to many currently used antibiotics^1–4^, with the danger that the treatment of infected patients/animals will become increasingly difficult. Thus, searching for new drug targets addressing these complex issues is of stringent relevance.

Carbonic anhydrases (CAs, EC 4.2.1.1) are ubiquitous metalloenzymes in all kingdoms^5–7^. They catalyze the reversible hydration of CO_2_ with formation of bicarbonate and protons, converting thus efficiently two neutral molecules (CO_2_ and H_2_O) in a weak base (bicarbonate) and a very strong acid (H^+^ ions)^8–10^. For this reason, in most organisms investigated so far, from simple (such as bacteria and archaea) to complex ones (plants, animals etc.) these enzymes are involved in pH regulation as well as several crucial metabolic pathways^5–7^ ^,^ ^11–17^. At least seven distinct CA genetic families are known to date (α-, β-, γ-, ζ-, η- and θ-CAs)^5–7^, and their diffusion and physiological roles have been investigated in details mainly in vertebrates, including humans, who posses only α-CAs, but with quite a large number of isoforms (15 CA isoforms are known in humans, human carbonic anhydrase (hCA) I-XIV, with two V-type ones, CA VA and VB)^5^. The investigation of CAs belonging to other classes, such as those found in bacteria, is on the other hand a rather recent field, although notable advances were registered in the last years^5–7^ ^,^ ^13^ ^,^ ^18^ ^,^ ^19^. CA inhibitors (CAIs) belonging to many diverse chemotypes and possessing a wealth of inhibition mechanisms^5–7^ are clinically used for the management of a variety of disorders, including edema, epilepsy, glaucoma, obesity, hypoxic tumors, neuropathic pain and arthritis^5–7^ ^,^ ^11–17^. On the other hand, CA activators (CAAs) started to be investigated in detail only in the last two decades, after the CA activation mechanism has been explained by one of our groups^8–10^.

Indeed, CAAs have been demonstrated to participate in the CA catalytic cycle^8^, which is shown schematically in Equations (1) and (2) below:

(1) $${\text{EZn}}^{2+}–{\text{OH}}^{-}+{\text{CO}}_{2}\Leftrightarrow {\text{EZn}}^{2+}–{\text{HCO}}_{3}^{-}\overset{H_{2}O}{\Leftrightarrow }{\text{EZn}}^{2+}–{\text{OH}}_{2}+{\text{HCO}}_{3}^{-}$$

(2) $${\text{EZn}}^{2+}–{\text{OH}}_{2}\Leftrightarrow {\text{EZn}}^{2+}–{\text{HO}}^{-}+H^{+}$$

In the first step, a zinc-bound hydroxide species of the enzyme with a strong nucleophilicity, attacks the CO_2_ substrate, which is weakly bound in a hydrophobic pocket nearby, being optimally orientated for the hydration reaction to occur by the attack of the zinc hydroxide nucleophile (Equation 1)^8^. In the next step of the process, the formed bicarbonate in the hydration reaction is replaced by an incoming water molecule, leading to the formation of an acidic enzymatic species, EZn^2+^—OH_2_ (Equation 1). In order to regenerate the zinc hydroxide species, a proton must be transferred from the Zn(II)-bound water molecule to the external medium (Equation 2). This is also the rate-determining step of the entire catalytic cycle^8–10^. In the presence of activators (A in Equation 3), this rate-determining step is facilitated by an additional proton release pathway, which involves the activator A bound within the enzyme active site. It should be noted that all CAAs known to date possess in their molecule protonatable moieties of the amine, carboxylate or imidazole type, with pK_a_ values in the range of 5–8^8–10^.

(3) $$\begin{array}{c} {\text{EZn}}^{2+}–{\text{OH}}_{2}+A\Leftrightarrow \left[{\text{EZn}}^{2+}–{\text{OH}}_{2}-A\right]\Leftrightarrow \left[{\text{EZn}}^{2+}–{\text{HO}}^{-}{-\text{AH}}^{+}\right]\Leftrightarrow {\text{EZn}}^{2+}–{\text{HO}}^{-}+{\text{AH}}^{+}\\ \text{enzyme}\;-\;\text{activator}\;\text{complexes} \end{array}$$

By the formation of enzyme-activator complex (Equation 3), the proton transfer reaction becomes intramolecular, and is thus more rapid compared with the intermolecular process in which for example buffer molecules participate^8–10^ ^,^ ^20^. The enzyme-activator complexes were thoroughly characterized for α-CAs of human origin, such as hCA I and II, by means of kinetic and X-ray crystallographic techniques, which allowed the identification of the activator-binding site within the CA cavity^8^ ^,^ ^9^ ^,^ ^21–29^. However, CAA research involving various activators has been relatively neglected compared with that of CAIs, with only a relatively limited number of studies focusing on activators of bacterial CAs^29^. Recently, it has been demonstrated that such activators may have pharmacological applications for enhancing cognition, in the management of CA deficiencies, for therapy memory and for obtaining artificial tissues a well as for investigating the effects of endogenous amino acids and amines on CA activity^30^.

Here we report the first activation study of the CAs investigated so far in the genome of the two pathogenic bacteria mentioned earlier. Indeed, *B. suis* encodes for two β-CAs, BsuCA1 and BsuCA 2^18^, whereas in the genome of *F. tularensis* only one such enzyme is present (again belonging to the β-CA class, FtuCA)^18^ which were cloned and characterized by some of us earlier^18^. The inhibition of these enzymes with sulfonamide, anions and other derivatives was reported earlier^31–33^, but no activation studies of the two enzymes are available in the literature. Here we report the first activation study of BsuCA 1 and FtuCA with a panel of amino acids and amines, known to act as CAAs for enzymes belonging to various other genetic families^29–34^.

## 2. Materials and methods

### 2.1. Materials

Amino acids and amines **1–24** were commercially available, highest purity reagents from Sigma-Aldrich, Milan, Italy. BsuCA 1 and FtuCA were recombinant proteins produced as reported earlier by our groups^18^.

### 2.2. CA enzyme activation assay

An Sx.18Mv-R Applied Photophysics (Oxford, UK) stopped-flow instrument has been used to assay the catalytic activity of various CA isozymes for CO_2_ hydration reaction^20^. Phenol red (at a concentration of 0.2 mM) was used as indicator, working at the absorbance maximum of 557 nm, with 10 mM Tris (pH 8.4) as buffer, and 0.1 M Na_2_SO_4_ (for maintaining constant ionic strength, which is not inhibitory against these enzymes^18^), following the CA-catalyzed CO_2_ hydration reaction for a period of 10 s at 25 °C. The CO_2_ concentrations ranged from 1.7 to 17 mM for the determination of the kinetic parameters and activation constants. For each activator at least six traces of the initial 5–10% of the reaction have been used for determining the initial velocity. The uncatalyzed rates were determined in the same manner and subtracted from the total observed rates. Stock solutions of activators (10 mM) were prepared in distilled-deionized water and dilutions up to 1 nM were done thereafter with the assay buffer. Activator and enzyme solutions were pre-incubated together for 15 min (standard assay at room temperature) prior to assay, in order to allow for the formation of the E–A complex. The activation constant (*K*_A_), defined similarly with the inhibition constant *K*_I_, can be obtained by considering the classical Michaelis–Menten equation (Equation 4), which has been fitted by non-linear least squares by using PRISM 3:

(4) $$v=v_{\text{max}}/\left\{1+K_{M}/\left[S\right]\left(1+{\left[A\right]}_{f}/K_{A}\right)\right\},$$

where [*A*]_f_ is the free concentration of activator.

Working at substrate concentrations considerably lower than *K*_M_ ([*S*] ≪*K*_M_), and considering that [*A*]_f_ can be represented in the form of the total concentration of the enzyme ([*E*]_t_)and activator ([*A*]_t_), the obtained competitive steady-state equation for determining the activation constant is given by Equation (5):

(5) $$v=v_{0}.K_{A}/\{K_{A}+({\left[A\right]}_{t}-0.5\{([A{]}_{t}+{\left[E\right]}_{t}+K_{A})-{([A{]}_{t}+{\left[E\right]}_{t}+K_{A})}^{2}-4[A{]}_{t}.{\left[E\right]}_{t}{)}^{1/2}\}\},$$

where *v*_0_ represents the initial velocity of the enzyme-catalyzed reaction in the absence of activator^21–29^. The results obtained with this type of kinetic assay are in excellent agreement with those obtained by use of other independent methods, including native mass spectrometry and fluorescence spectroscopy^35^.

## 3. Results and discussion

**Figure 1.**
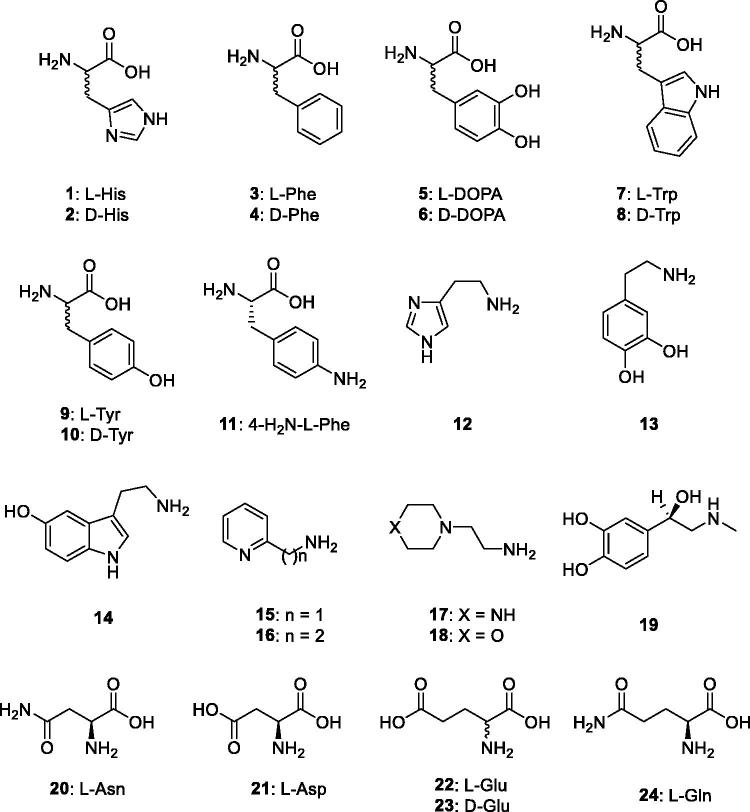
Amino acids and amines **1–24** investigated as activators.

Natural and non-natural amino acids and amines **1–24** were included among the investigated compounds as activators of the two bacterial β-CAs investigated here (Figure 1). These compounds were employed for investigations as CAAs against many classes of CAs, including the bacterial, archaeal and mammalian ones, as mentioned earlier^8–10^ ^,^ ^21–29^. The salient feature of these compounds is the presence of protonatable moieties of the amine, carboxylate or imidazole type, which makes them appropriate for participating in the proton shuttling processes between the active site and the reaction medium, as described by Equation (3).

Data of Table 1 demonstrate that the two bacterial β-CAs investigated here shows a more effective CO_2_ hydrase activity compared with hCA I, a widely spread isoform in humans^8^. Considering the *k*_cat_, BsuCA 1 is 3.2-times more effective as a catalyst for CO_2_ hydration compared with hCA I, whereas FtuCA is even more active, being 4.9-times more effective compared with the human enzyme. On the other hand, the Michaelis constants (*K*_M_) of the bacterial enzymes are higher compared with the two human enzymes showed in Table 1 for comparison reasons. In fact the Michaelis constant of hCA I and II is in the range of 4.0–9.3 mM, whereas for the bacterial enzymes the *K*_M_ values are in the range of 11.0–16.4 mM, denoting that CO_2_ has a lower affinity for the bacterial enzymes than for the human ones hCA I/II. We have chosen L-Trp for performing detailed kinetic measurements because this amino acid was a rather effective activator for both enzymes investigated here (as well as the human isoforms hCA I and II used as standard enzymes in such experiments – see Section 3). In the presence of 10 µM L-Trp as activator, the *K*_M_ of BsuCA 1/FtuCA remained unchanged (data not shown) but the *k*_cat_ was 4.60 times higher than in the absence of the activator for BsuCA 1, and 1.60-times higher for FtuCA, respectively (Table 1). This situation, in which the *K*_M_ does not depend on the presence of an activator and *k*_cat_ is strongly affected by the activator, has been observed for all CAs investigated to date, belonging to all known CA genetic families. This strongly indicates that the CA activation mechanism is similar for all enzyme classes, involving facilitation of the proton transfer process by the activator molecule bound within the enzyme active site in the enzyme-activator complex^8–10^. It should be also noted that L-Trp is a much more effective activator for BsuCA 1 than for FtuCA (see Section 3).

**Table 1.** Activation of hCA isozymes I, II and BsuCA 1/FtuCA, with L-Trp, at 25 °C, for the CO_2_ hydration reaction^20^.

Isozyme	*k*_cat_ (s^−1^)^a^	*K*_M_ (mM)^a^	(*k*_cat_)_L-Trp_ (s^−1^)^b^	*K*_A_ (μM) L-Trp^c^
hCA I^d^	2.0 × 10^5^	4.0	3.4 × 10^5^	44
hCA II^d^	1.4 × 10^6^	9.3	4.9 × 10^6^	27
BsuCA 1^e^	6.4 × 10^5^	16.4	25.9 × 10^5^	1.25
FtuCA^e^	9.8 × 10^5^	11.0	15.7 × 10^5^	34.1

a Observed catalytic rate without activator. *K*_M_ values in the presence and the absence of activators were the same for the various CAs (data not shown).

b Observed catalytic rate in the presence of 10 μM activator.

c The activation constant (*K*_A_) for each enzyme was obtained by fitting the observed catalytic enhancements as a function of the activator concentration^21–29^. Mean from at least three determinations by a stopped-flow, CO_2_ hydrase method^20^. Standard errors were in the range of 5–10% of the reported values (data not shown).

d Human recombinant isozymes, from Supuran^8^a.

e Bacterial recombinant enzyme, this work.

In Table 2 the activation constants (*K*_A_s) of the bacterial β-CAs investigated here (BsuCA 1 and FtuCA) and of the human isoforms hCA I and II, with the amino acid and amine derivatives **1–24** (Figure 1) are shown. The human CA activation data are reported for comparison reasons.

**Table 2.** Activation constants of hCA I, hCA II and the bacterial β-CAs investigated here with amino acids and amines **1–24**, by a stopped-flow CO_2_hydrase assay^20^.

No.	Compound	*K*_A_ (μM)^a^			
		hCAI^b^	hCAII^b^	BsuCA1^c^	FtuCA^c^
1	L-His	0.03	10.9	1.76	40.7
2	D-His	0.09	43	12.3	78.3
3	L-Phe	0.07	0.013	1.16	69.1
4	D-Phe	86	0.035	1.21	75.0
5	L-DOPA	3.1	11.4	2.07	>100
6	D-DOPA	4.9	7.8	2.34	44.8
7	L-Trp	44	27	1.25	34.1
8	D-Trp	41	12	13.7	30.5
9	L-Tyr	0.02	0.011	1.38	>100
10	D-Tyr	0.04	0.013	0.95	>100
11	4-H_2_N-L-Phe	0.24	0.15	1.18	>100
12	Histamine	2.1	125	3.71	>100
13	Dopamine	13.5	9.20	1.54	>100
14	Serotonin	45	50	4.26	>100
15	2-Pyridyl-methylamine	26	34	1.62	46.3
16	2-(2-Aminoethyl)pyridine	13	15	5.20	>100
17	1-(2-Aminoethyl)-piperazine	7.4	2.30	43.1	51.8
18	4-(2-Aminoethyl)-morpholine	0.14	0.19	9.56	>100
19	L-Adrenaline	0.09	96	0.70	>100
20	L-Asn	11.3	>100	7.38	>100
21	L-Asp	5.20	>100	6.82	>100
22	L-Glu	6.43	>100	9.36	9.13
23	D-Glu	10.7	>100	1.39	>100
24	L-Gln	>100	>50	3.21	45.7

a Mean from three determinations by a stopped-flow, CO_2_ hydrase method^20^. Standard errors were in the range of 5–10% of the reported values (data not shown).

b Human recombinant isozymes, stopped flow CO_2_ hydrase assay method^26^.

c Bacterial enzyme, this work.

Data of Table 2 show the following interesting features for the activation of BsuCA 1 and FtuCA with amino acid and amine derivatives **1–24**:

- The enzyme from *B. suis*, BsuCA 1 was generally rather sensitive to activation with amino acids and amines of type **1–24**, with activation constants in the range of 0.70–43.1 µM. On the contrary, 13 out of the 24 compounds investigated here were inactive as activators of FtuCA (*K*_A_ > 100 µM), only one compounds had a *K*_A_ < 10 µM (L-Glu, **22**), and most of the investigated active compounds showed a weak activating effect, with *K*_A_s ranging between 30.5 and 78.3 µM. Thus, only L-Glu may be considered a an effective FtuCA activator. Small changes in its scaffold, such as the transformation of the COOH moiety in CONH_2_ lead to a drastic loss of activity (compound **24**), whereas the D-enantiomer **23** was devoid of activity (Table 2). Thus, the structure-activity relationship will be discussed for the activation of BsuCA 1, as the profile for activating FtuCA with these compounds is quite flat and the activation effects are modest or absent.
- The most effective BsuCA 1 activators were L-His, L-/D-Phe, L-/D-DOPA, L-Trp, L-/D-Tyr, 4-amino-L-Phe, dopamine, 2-pyridyl-methylamine, L-adrenaline, D-Glu and L-Gln. These compounds showed activation constants in the range of 0.70–3.21 µM. Generally the L-amino acid derivatives were more efficient BsuCA 1 activators compared with the corresponding D-enantiomers, but some exceptions from this rule were also encountered, with D-Tyr and D-Glu being better activators than the corresponding L-amino acids (Table 2). Rather unexpectedly, the most efficient activator was L-adrenaline, with a *K*_A_ of 0.70 µM.
- Slightly less effective BsuCA 1 activators were D-His, D-Trp, histamine, heterocyclic amines **16–18**, L-Asn, L-Asp and L-Glu, which had activation constants in the range of 3.71–43.1 µM. Only compound **17** can be considered a weak activator against this isoform (*K*_A_ of 43.1 µM), with all the other ones being moderate activators.
- The activation profile of the two bacterial enzymes investigated here is very different from the ones of the two human isoform hCA I and II showed in Table 1 as reference enzymes. Furthermore, as mentioned above, the two bacterial enzymes differ considerably in their interaction with these activators.

## 4. Conclusions

The first activation study of the β-class CA enzymes from the pathogenic bacteria *B. suis* and *F. tularensis* is reported here. A panel of 24 amino acid and amine derivative were included in the study. BsuCA 1 was sensitive to activation with amino acids and amines, which showed activation constants in the range of 0.70–43.1 µM. The most effective BsuCA 1 activators were L-adrenaline and D-Tyr (*K*_A_s of 0.70–0.95 µM). L-His, L-/D-Phe, L-/D-DOPA, L-Trp, L-Tyr, 4-amino-L-Phe, dopamine, 2-pyridyl-methylamine, D-Glu and L-Gln showed activation constants in the range of 0.70–3.21 µM. FtuCA was not sensitive to activation with many of the investigated compounds (*K*_A_ > 100 µM), and only L-Glu had a *K*_A_ < 10 µM. Most of the investigated active compounds showed a weak activating effect against FtuCA, with *K*_A_s ranging between 30.5 and 78.3 µM, such a L-/D-His, L-/D-Phe, L-/D-Trp, 2-pyridyl-methylamine. It should be noted that many of the investigated amino acid and amines are present in rather high concentrations in many tissues in vertebrates, and their role in the pathogenicity of the two bacteria is poorly understood. Our study may thus bring some insights in the intricate processes connected with the invasion and pathogenic effects of intracellular bacteria when attacking their hosts.
